# Coexistence of endobronchial aspergilloma, pulmonary embolism, and lung cancer: a case report

**DOI:** 10.3389/fonc.2025.1724471

**Published:** 2025-12-04

**Authors:** Chen Zhu, Zhanshi Jiang, Hui Yu, Ying Gu

**Affiliations:** 1 Department of Respiratory and Critical Care Medicine, Yantai Yuhuangding Hospital, Yantai, Shandong, China; 2 Department of Respiratory and Critical Care Medicine, Yantai Affiliated Hospital of Binzhou Medical University, Yantai, Shandong, China

**Keywords:** endobronchial aspergilloma, lung cancer, pulmonary embolism, coexistence, fiberoptic bronchoscopy

## Abstract

**Background:**

Endobronchial aspergilloma (EBA) represents an uncommon manifestation of pulmonary aspergillosis that may co-occur with other significant respiratory pathologies, notably lung cancer and pulmonary embolism (PE), complicating diagnosis and management.

**Case presentation:**

An 83-year-old male with a history of benign prostatic hyperplasia surgery presented with chest pain. Computed tomography angiography (CTA) of the pulmonary arteries revealed both PE and a left hilar mass. Fiberoptic bronchoscopy disclosed a yellow, mushroom-like lesion, with histological examination confirming squamous cell carcinoma. Subsequent bronchoalveolar lavage culture was positive for Aspergillus flavus, confirming the diagnosis of EBA. Despite initiated treatments, the patient declined further therapeutic interventions and succumbed one month following presentation.

**Conclusion:**

This case underscores the rare coexistence of EBA, lung cancer, and PE, highlighting considerable diagnostic challenges and the need for integrated multidisciplinary management strategies in such complex presentations.

## Background

Endobronchial aspergilloma (EBA) represents an uncommon form of pulmonary aspergillosis characterized by the formation of a fungal mass within the bronchial lumen. It typically develops in patients with pre-existing airway abnormalities, such as those resulting from tumors, foreign bodies, tuberculosis, or chronic obstructive pulmonary disease (COPD) (1, 2). These structural alterations promote airflow stagnation, creating a conducive environment for the colonization of *Aspergillus* species—most commonly *Aspergillus fumigatus* (3), along with rarer pathogens such as Fusarium in immunocompromised hosts (4). Although less frequently documented, *Aspergillus flavus* has also been identified in a limited number of EBA cases. Epidemiologically, EBA is more prevalent among immunocompromised individuals, including those with malignancies, prolonged corticosteroid use, or other immunosuppressive conditions. The co-occurrence of EBA and lung cancer is uncommon and poses diagnostic difficulties due to overlapping clinical manifestations. The pathogenesis of endobronchial aspergilloma (EBA), classically defined as the saprophytic colonization of Aspergillus in pre-existing bronchial cavities or stenotic areas, involves localized fungal growth within the bronchial lumen that is facilitated by such airway obstruction or structural compromise (5). Pulmonary embolism (PE), a well-recognized complication in lung cancer patients, may further obscure the clinical picture, potentially delaying the diagnosis of both EBA and underlying malignancy. In this case of an 83-year-old man who presented with chest pain following prostate surgery. Through computed tomography pulmonary angiography (CTPA), multidisciplinary assessment, and fiberoptic bronchoscopy, the patient was diagnosed with concurrent EBA, PE, and lung cancer. A review of the existing literature confirms that the simultaneous presence of these three conditions is rare.

## Case presentation

**Figure 1:**
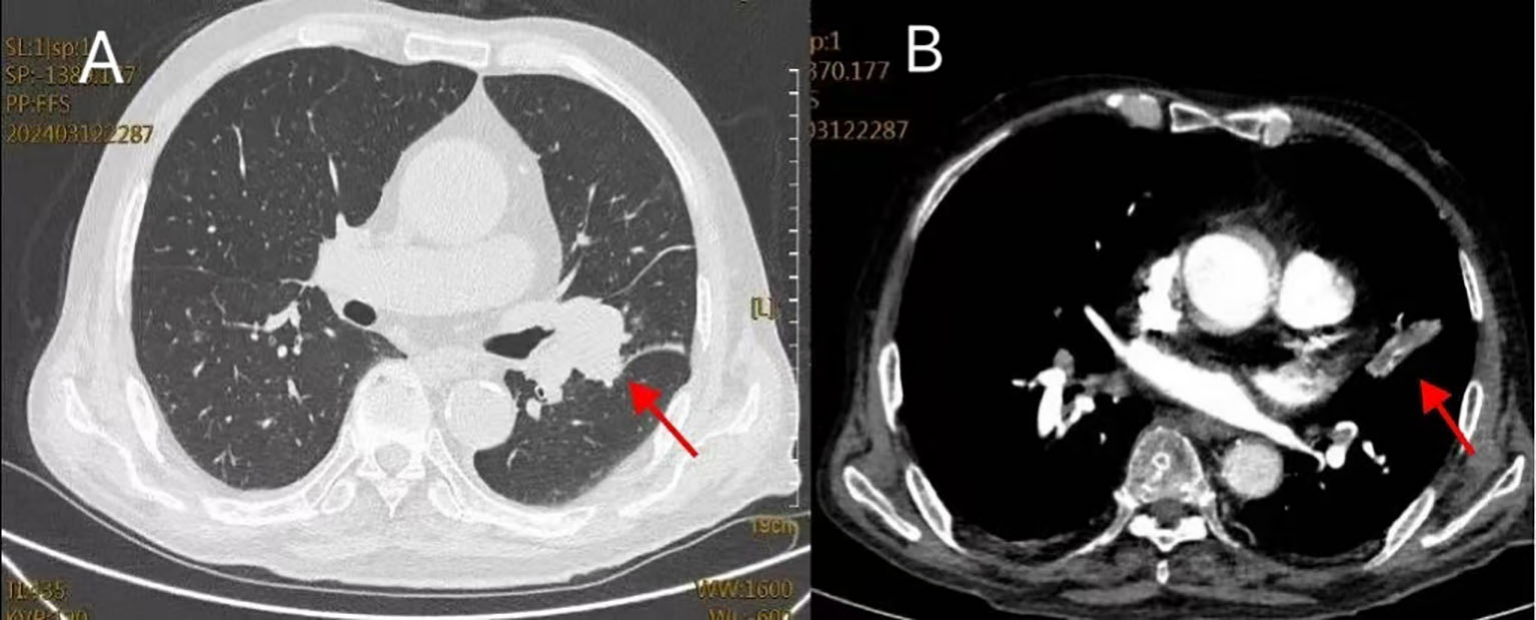
**(A)** (Lung window): Mass in the left hilum with surrounding obstructive pneumonitis. **(B)** (Mediastinal window): Multiple filling defects in bilateral pulmonary arteries, consistent with pulmonary embolism; the left hilar mass is also seen.

On March 11, 2024, an 83-year-old man presented with a three-month history of sharp, paroxysmal right-sided chest pain. His medical history included hypertension for 10 years, a significant 40-pack-year smoking history and recent surgery for benign prostatic hyperplasia. Physical examination demonstrated diminished breath sounds on the left side and coarse breath sounds on the right. Laboratory tests revealed leukocytosis (10.45×10^9^/L; reference range: 3.5-9.5×10^9^/L), elevated inflammatory markers including C-reactive protein (CRP 69.18 mg/L; reference range: <5 mg/L) and serum amyloid A (SAA 141.02 mg/L; reference range: <10 mg/L), and increased D-dimer (5.54 mg/L; reference range: <0.5 mg/L). The tested tumor markers showed mild elevations, including neuron-specific enolase (NSE) at 30.6 ng/mL (reference range: <16.3 ng/mL), cytokeratin 19 fragment (CYFRA 21-1) at 4.67 ng/mL (reference range: <3.3 ng/mL), and carbohydrate antigen 72-4 (CA72-4) at 22.3 U/mL (reference range: <6.9 U/mL). Serum Aspergillus IgG level was within normal limits (<31.25 AU/mL; reference range: <80 AU/mL).Given the elevated D-dimer level, a vascular ultrasound was performed and confirmed the presence of right posterior tibial and left intermuscular venous thrombosis, while CTPA showed multiple pulmonary emboli with a left hilar mass (**Figure 1**). The final diagnoses were pulmonary mass, pulmonary embolism (low risk as per the Pulmonary Embolism Severity Index [PESI] score of 32, class I), and deep vein thrombosis.

**Figure 2:**
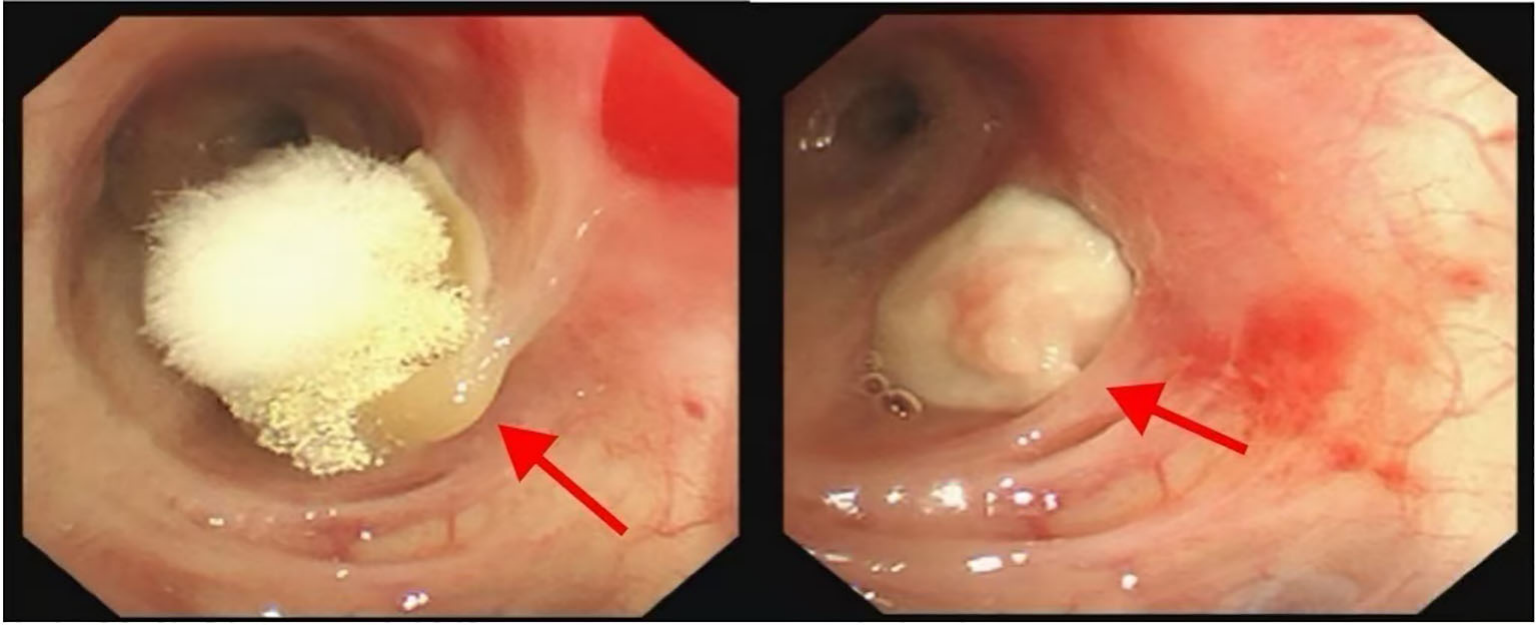
The fiberoptic bronchoscope revealed a mass at the opening of the left lung tongue segment, covered with a yellow, mushroom-like substance.

**Figure 3:**
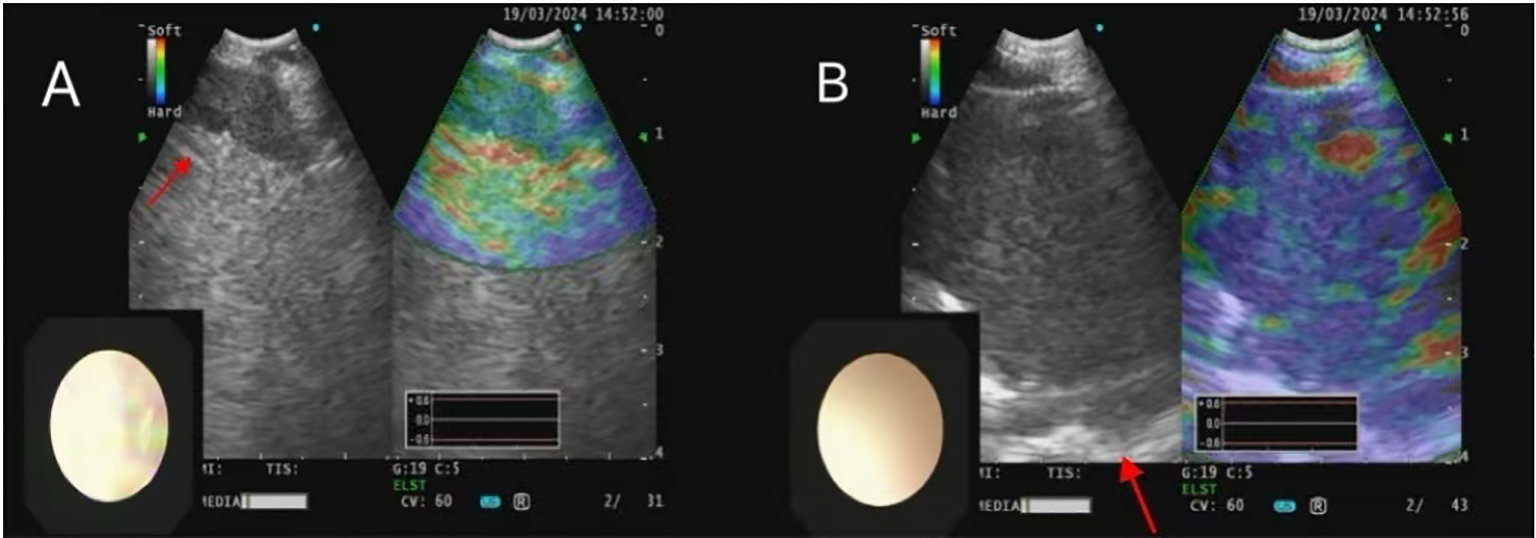
**(A)** EBUS showed enlarged lymph nodes in the 7 group; **(B)** EBUS showed enlarged lymph nodes in the 11L group, with the largest measuring 3.32× 3.09cm.

**Figure 4:**
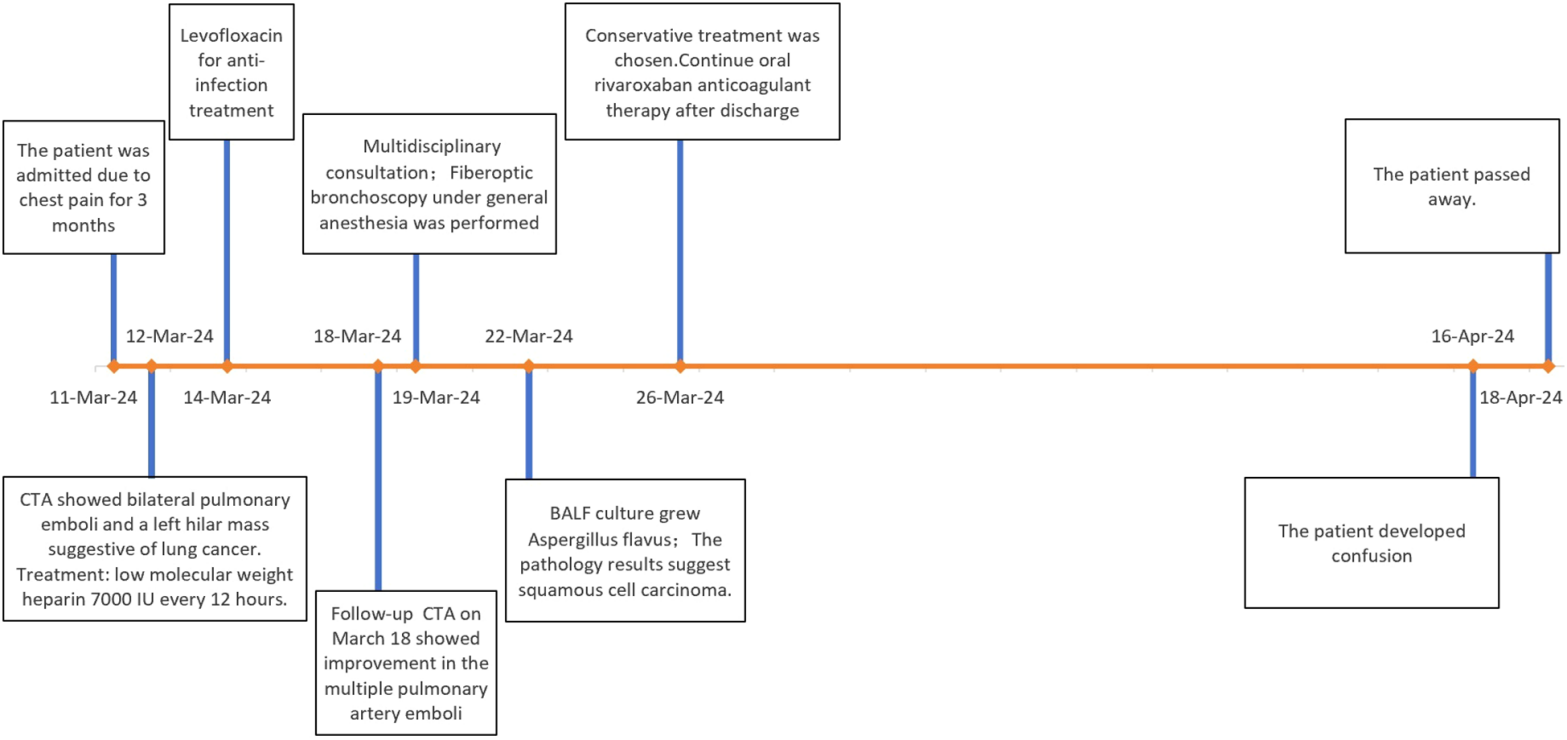
The clinical treatment course.

Therapeutic anticoagulation was started with low molecular weight heparin (7000 IU subcutaneously every 12 hours). Fiberoptic bronchoscopy was scheduled to evaluate the pulmonary mass. For pathological diagnosis of suspected lung cancer, multidisciplinary consensus recommended proceeding with bronchoscopy under laryngeal mask anesthesia, involving 24-hour Low Molecular Weight Heparin(LMWH) withdrawal and continuous hemodynamic monitoring, with postoperative Intensive Care Unit(ICU) care Fiberoptic bronchoscopy revealed a yellow, mushroom-like mass obstructing the left lingular orifice(**Figure 2**). Procedural interventions included bronchoalveolar lavage, brushing, and biopsy. EBUS demonstrated enlarged lymph nodes at stations 7 and 11L (largest: 3.32×3.09 cm) (**Figure 3**). Histopathological examination, supplemented by Grocott methenamine silver (GMS) and periodic acid-Schiff (PAS) staining, confirmed squamous cell carcinoma while demonstrating no evidence of fungal invasion. This finding is consistent with the saprophytic nature of an aspergilloma and, when integrated with the direct bronchoscopic visualization of a mycetoma and compelling microbiological evidence from BALF (including culture of Aspergillus flavus and a markedly elevated galactomannan index of 6.68(reference positive>1.0), collectively supported the diagnosis of EBA. Imaging studies, including contrast-enhanced abdominal CT, cranial MRI, and a whole-body bone scan, demonstrated metastases to the liver, brain, and bones (with destruction), compatible with a diagnosis of Stage IVB (cT4N3M1c) lung cancer. The patient died approximately one month post-discharge, after refusing treatment and subsequently developing progressive confusion, with the presumed cause of death being multiple organ failure from underlying systemic progression of his stage IVB lung cancer and associated brain metastases. The clinical treatment course is depicted in **Figure 4**.

## Discussion

The pathogenesis of aspergilloma, a saprophytic fungal aggregation, typically hinges on colonization of pre-formed pulmonary cavities (6). When this process occurs within the bronchial tree, it is termed endobronchial aspergilloma (EBA), a rare entity often associated with structural compromise or immune deficiency (7–11). It is noteworthy that cavitary or necrotic lung cancers, particularly squamous cell carcinoma, are recognized predisposing factors (6). This established link is central to understanding our patient’s presentation. We postulate that the left hilar squamous cell carcinoma acted as the primary lesion, whose necrotic core and obstructive nature created a localized, immunocompromised cavity-like environment. This setting was directly conducive to the colonization by *Aspergillus flavus* and the formation of the EBA, whose obstructive presence ultimately risked masking the true underlying diagnosis of malignancy (2). The diagnosis of EBA over IPA in this case was established by the presence of an endobronchial mass on imaging—distinct from IPA patterns like the halo sign—and the histopathologic absence of fungal invasion on special stains (5). Nevertheless, while a positive BALF galactomannan and culture are more common in IPA, the specific context of the endobronchial lesion in our patient makes EBA the more fitting diagnosis (9). Despite a normal serum *Aspergillus* IgG level, the significantly elevated BALF galactomannan and positive *Aspergillus flavus* culture indicated localized fungal infection. This serological profile likely resulted from confined antigen release alongside a systemically impaired humoral immunity caused by the advanced cancer. The present case illustrates a highly uncommon triad of pathophysiological processes: endobronchial aspergilloma, squamous cell carcinoma, and PE. Beyond their mere coexistence, this case suggests potential synergistic interactions among these conditions, which likely contributed to the diagnostic challenges and rapid clinical deterioration.

The sequence of events in our patient underscores a plausible pathogenic link between lung cancer and EBA. The centrally located squamous cell carcinoma likely served as the primary instigator, causing critical airway obstruction. This obstruction, in turn, created a local microenvironment characterized by mucus retention, impaired mucociliary clearance, and airflow stagnation—ideal conditions for the colonization and proliferation of Aspergillus species (8, 12, 13). Furthermore, the tumor microenvironment (TME) of lung cancer is increasingly recognized for its immunosuppressive properties. As evidenced by recent studies, the TME can dampen effector T-cell responses and alter local immune surveillance (14), thereby facilitating fungal colonization without immediate tissue invasion. The hypoxic and acidic conditions within the TME may have further supported the survival of *Aspergillus flavus* (15). The identification of *Aspergillus flavus* is critical due to its distinct antifungal susceptibility, particularly a common resistance to itraconazole, unlike A. fumigatus. This directly influences empiric therapy, guiding clinicians away from potentially ineffective treatments. In this context, the EBA was not an independent entity but rather a secondary complication fueled by the obstructive and immunomodulatory effects of the underlying malignancy. Concurrently, the patient’s course was complicated by persistent PE despite therapeutic anticoagulation. This clinical observation underscores the profound prothrombotic state associated with advanced malignancy, particularly lung cancer. Tumor-derived exosomes and procoagulant factors are known to activate platelets and the coagulation cascade, leading to venous thromboembolism that is often refractory to standard anticoagulation regimens (16, 17). While a direct causal link is difficult to establish, it is also conceivable that the local and systemic inflammatory response elicited by the EBA (18, 19) may have further exacerbated this hypercoagulable state, creating a vicious cycle of thrombosis and inflammation. A critical aspect of this case’s management was the decision-making regarding antifungal therapy. Although Aspergillus flavus was confirmed by BALF culture with a markedly elevated galactomannan index, systemic antifungal therapy was not initiated following the diagnosis. This decision was primarily guided by the patient’s dismal prognosis from stage IVB lung cancer with multisystem metastases, his overall poor performance status, and, ultimately, his family’s preference to forego aggressive interventions focused on comfort care. This highlights a common yet under-discussed dilemma in palliative oncology: the management of opportunistic infections in the terminal phase, where the benefits of treatment must be carefully weighed against the goals of care and potential burdens on the patient. This management challenge is further compounded when considering that conventional anticancer treatments like chemotherapy or radiotherapy could have exacerbated the patient’s immunosuppression, potentially converting the non-invasive EBA into invasive aspergillosis (20). Therefore, while bronchoscopic intervention combined with antifungal therapy represents a viable and reported strategy for managing obstructive EBA (21–23), it was deemed inappropriate in this specific palliative context. Although this patient did not receive antifungal therapy due to poor prognosis, for EBA patients with active malignancy requiring intervention, treatment strategies should be carefully weighed within a multidisciplinary framework. The primary concern is drug interactions—azole agents such as voriconazole may significantly increase the hematological or organ toxicity of chemotherapeutic drugs by inhibiting the cytochrome P450 system (24). In such cases, echinocandins with fewer interactions may be prioritized, and monitoring of liver/kidney function, complete blood count, and azole drug concentrations is essential to balance efficacy and toxicity (25). While no specific guidelines exist for EBA, as a localized infection, its treatment duration may be shorter than that for invasive aspergillosis, though more evidence is needed to form a consensus. This study has several inherent limitations due to its nature as a single case report. The findings are descriptive and cannot be generalized. However, its novelty as the first reported instance of this clinical triad provides valuable insight, highlighting the importance of integrated diagnostics in complex oncology cases.

In conclusion, this case provides several critical insights for clinical practice. First, the discovery of an endobronchial aspergilloma should prompt an exhaustive search for an underlying malignant airway obstruction. Second, the management of cancer-associated thrombosis can be exceptionally challenging in the presence of advanced disease, and may not fully respond to anticoagulation. Finally, this case underscores the necessity of an individualized, multidisciplinary approach that integrates pulmonology, oncology, infectious disease, and palliative care from the outset. Such an approach is paramount for navigating the complex trade-offs between managing competing life-threatening conditions and aligning with patient-centered goals of care, especially when curative intent is no longer feasible.

## Ethical approval and informed consent

Written informed consent for publication of this case and the associated images was obtained from the patient’s son, as the patient had passed away. The retrospective analysis received approval from the Ethics Committee of Yantai Yuhuangding Hospital.

## Data Availability

The original contributions presented in the study are included in the article/supplementary material. Further inquiries can be directed to the corresponding authors.
